# Prevalence of pseudoexfoliation among patients at the Kolar district's tertiary care teaching Institute

**DOI:** 10.6026/9732063002001002

**Published:** 2024-09-30

**Authors:** Karnati Divija, Inchara Nagaraj

**Affiliations:** 1 Department of Ophthalmology, Sri Devaraj Urs Medical College, Tamaka Kolar, Karnataka, India

**Keywords:** Pseudoexfoliation, prevalence, IOP

## Abstract

The prevalence of pseudoexfoliation among patients seeking treatment at a teritiary care teaching institute of kolar district is of interest. This was a cross sectional observational study done at teritiary care center 3649 subjects underwent complete ophthalmic evaluation was done including history, visual acuity, slit lamp examination for cataract grading and posterior segment evaluation and IOP. Pseudoexfoliation Patients data were analyzed with respect to age, sex, IOP, cataract and optic neuropathy. 372subjects had pseudoexfoliation syndrome (10.1 %). There was a significant increase in prevalence with age more in age group of 61-70 years increase preponderance in males. 53 cases with PEX (14.2%) had high IOP, and 41 cases (11%) had PEX glaucoma. The prevalence of pseudoexfoliation syndrome was 10.1%. Raised IOP was seen in14.2 % of people with pseudoexfoliation and glaucoma was present in11% of people.

## Background:

Pseudoexfoliation syndrome, often known as PEX or PXF, is a systemic, age-related, elastic microfibrillopathy that presents with ocular system manifestations. [1] The first ever description of this disorder was in 1917 in a Thesis by Lindberg [2]. He had made a note of grayish material in the pupillary border in 50% of patients with glaucoma [2]. In 1926, the term "capsular glaucoma" was coined, as it was hypothesized that the material observed could have its origins in the lens anterior capsule [3]. However, by 1954, Dvorak-Theobald, proposed the term "pseudoexfoliation of the lens capsule" and differentiated it from true exfoliation of the lens capsule, noting deposits on the ciliary body and zonules as well as uncertainties regarding its origins. [4] Transmission electron microscopy ultra-structural studies have revealed the presence of PXF fibrils in the trabecular endothelium, corneal endothelium, pre-equatorial lens epithelium, non-pigmented ciliary epithelium, and vascular endothelial cells [5]. Autopsy tests have revealed the existence of PXF material in tissue samples from the kidneys, liver, heart, lungs, skin and cerebral meninges of the cerebellum and cerebrum [5]. Pseudoexfoliation material deposits on various structures of the anterior segment in the eye. The nature of this material is mostly fibrillar with fibers made up of microfibrils and coated with amorphous material [7]. Widespread extracellular matrix condition characterized by the formation of material in various intraocular and extraocular tissues that resembles aberrant basement membranes [1]. The trigger for the production of PEX material remains to be identified [4]. This entity must be recognized in order to identify potential catastrophic surgical complications during regular cataract surgery [3]. The worldwide prevalence of pseudoexfoliation (PXF) and pseudoexfoliation glaucoma (PXF) varies widely ranging from 0-80% with maximal prevalence in Scandinavian countries [6]. Pseudoexfoliation (PEX) syndrome is the most common identifiable cause of open angle glaucoma worldwide [10]. The prevalence of PXF based on hospital reports from India varies between 1.87% and 13.5%. Population-based studies from south India have recently reported the prevalence of PXF to be between 3.8% and 6.0% among persons aged 40 years [PXF is associated with inadequate dilatation and raises the chance of vitreous loss following cataract surgery as well as zonular dialysis [6]. PXF is crucial for the purpose of managing glaucoma and cataracts, [8]. Therefore it is of interest to report Pseudoexfoliation prevalence in Kolar district's tertiary care teaching institute.

## Materials & Methods:

## Study design:

A cross sectional observational study

## Source of data:

Records of patients who visit Ophthalmology OPD at R. L Jalappa Hospital, Kolar attached to Sri Devaraj Urs Medical College, Tamaka and Kolar

## Study duration:

August 2022 to 2023

## Inclusion criteria:

[1] Patients aged > 40 years of age

[2] Patients who are residents of kolar and chikkaballapur district for more than 6 months visiting RLJH

## Exclusion criteria:

Patients who had history of ocular trauma, uveitis history, vitro retinal surgery

## Data collection:

Data collected through the records of patients who attended ophthalmology OPD from January 2020 to July 2023 thoroughly. These records had complete demographic details of the patients including age, sex, address .complete history pertaining to history of present illness, past history, family history, Habits. From the records of the patients we collected details of complete ophthalmic evaluation including visual acuity, slit lamp examination for cataract grading, Pseudoexfoliation grading and posterior segment evaluation, Fundoscopic examination, Intraocular pressure. These important aspects of the history and examinations were noted down and subjected to analysis.

## Statistical methods:

Data entered into Microsoft excel data sheet and analyzed using SPSS 22 version software. Categorical data represented in the form of Frequencies and proportions. Chi-square is used as test of significance. Continuous data represented as mean and standard deviation. Independent t test used as test of significance to identify the mean difference P-value < 0.05 will be considered as statistically significant.

## Results:

## Prevalence:

**Figure 1:**
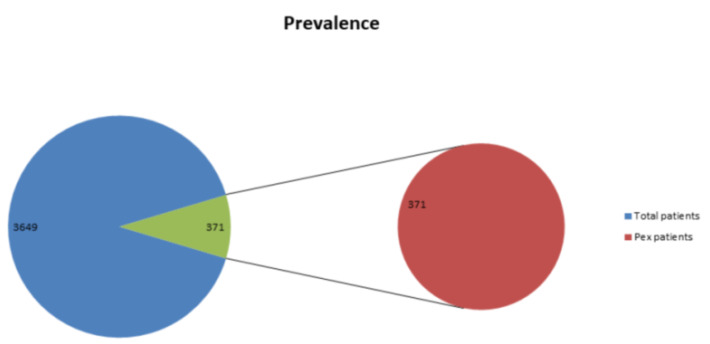
Prevalence of Pex

Data of 3649 patients were analyzed out of that 372 subjects had pseudoexfoliation syndrome (10.1 %)(Figure 1).

## Age:

**Figure 2:**
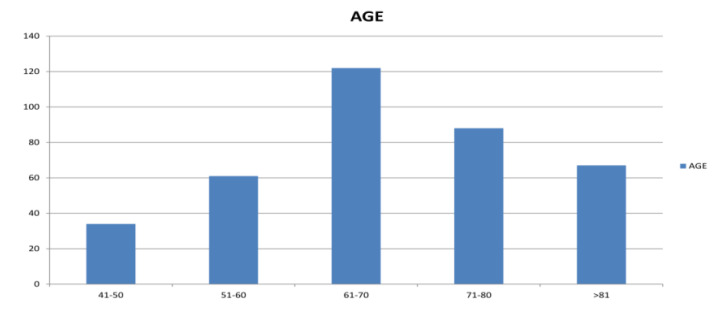
Age wise distribution of pex

The mean age of the patients was 66.2 ± 11.67 years. The most common age group of the patients was distributed between 61 and 70 years followed by 71 and 80 years. The distribution of patients in each age decade is presented (Figure 2).

## Sex:

There were 241 (67.08%) male and 131(32.92%) female patients. The overall distribution of PXF syndrome was significantly greater in males as compared to females and was statistically significant (P ≤ 0.00001). Among the patients diagnosed with PXF, the mean and were 67.3 ± 10.95 and 68 for men and 65.08 years for women, respectively.

## Socio-economic status:

**Figure 3:**
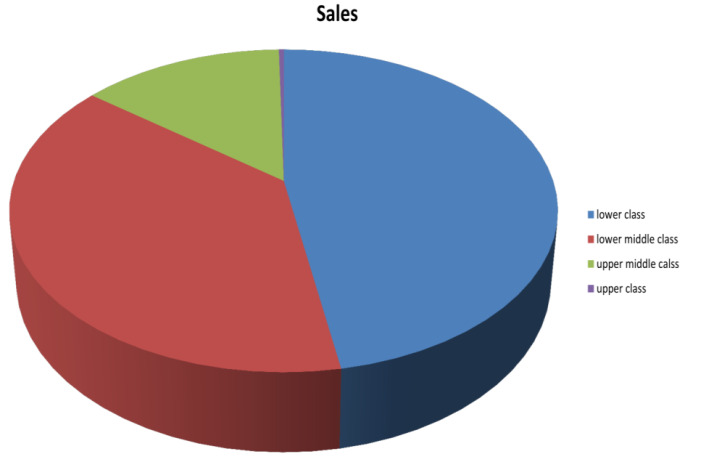
Distribution of pex among various socio economic groups

Of the 372 patients with PXF, there were 163 patients from the lower socio-economic class, 132 patients from the lower middle class, 49 patients from the upper middle class and 28 patients from the upper class. The overall prevalence was significantly higher in the lower socio-economic strata as compared to higher socio-economic strata and was statistically significant (P ≤ 0.00001)(Figure 3).

## Occupation:

**Figure 4:**
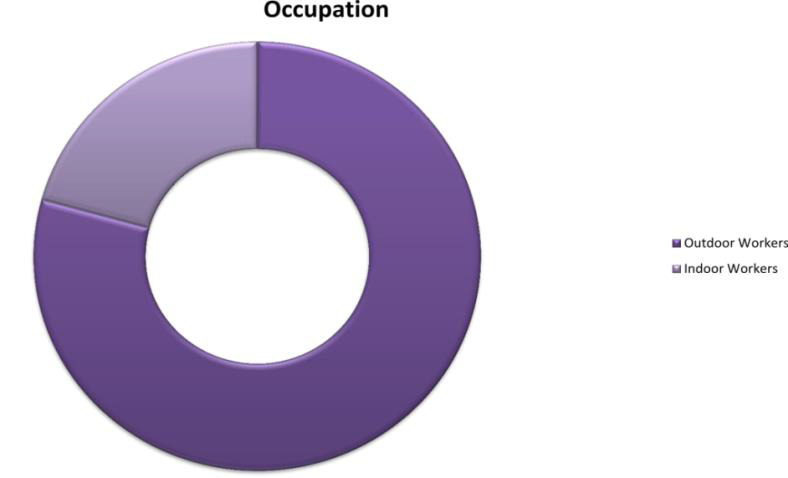
Occupational distribution of PEX

295 (79.2%) patients are outdoor workers such as daily laborers, agricultural workers (Figure 4).

## Systemic co-morbidities:

**Figure 5:**
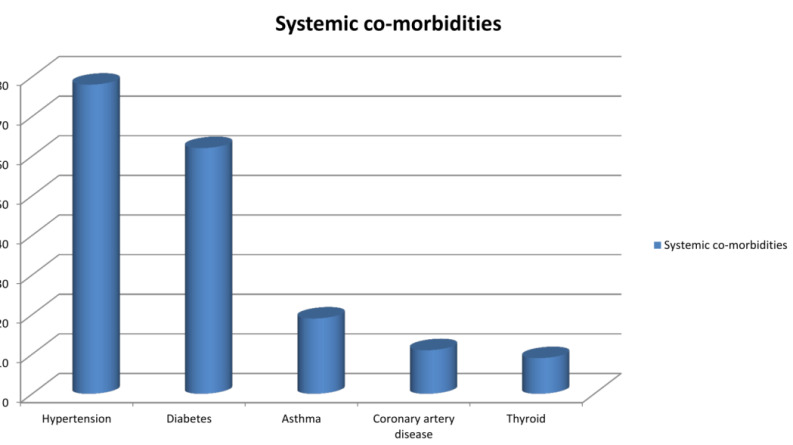
Distribution of PEX across various systemic comorbidities

Among the patients, hypertension was documented in 78 (20.9% )patients, diabetes mellitus in 62 ( 16.6% ) patients, asthma in 19 (5.1%) patients, coronary artery disease in 11 ( 2.9%) and thyroid disorders in 9 (2.4%)patients (Figure 5).

## Laterality:

**Figure 6:**
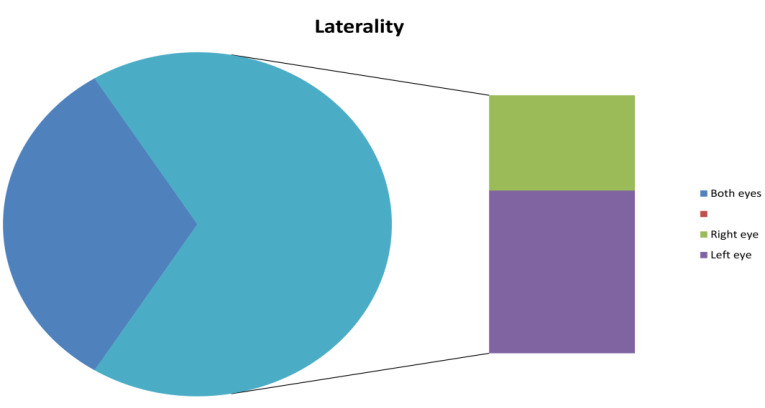
Distribution of PEX by Ocular Laterality

Of the 372, 93 (25%) were affected in the left eye and 159 (42.7%) were affected in the right eye. In 120 (32.2%) patients, the affliction was bilateral in nature (Figure 6).

## Location:

In the 372 eyes, the most common location of the PXF material was the pupillary margin in 221 (59.4%) eyes, the rest of the eye 151 (40.6%) eyes.

## Habits:

**Figure 7:**
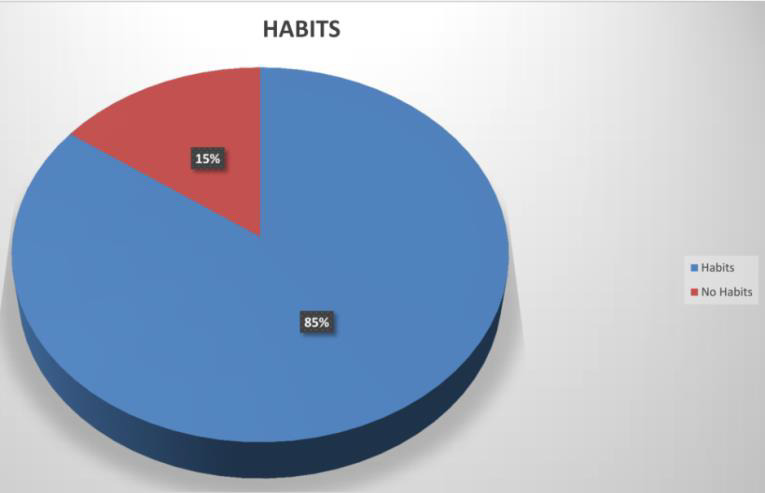
Distribution of PEX according to Habitual Factors

History of smoking and tobacco chewing are seen in 316 (85%) case (Figure 7).

## Ocular comorbidities:

**Figure 8:**
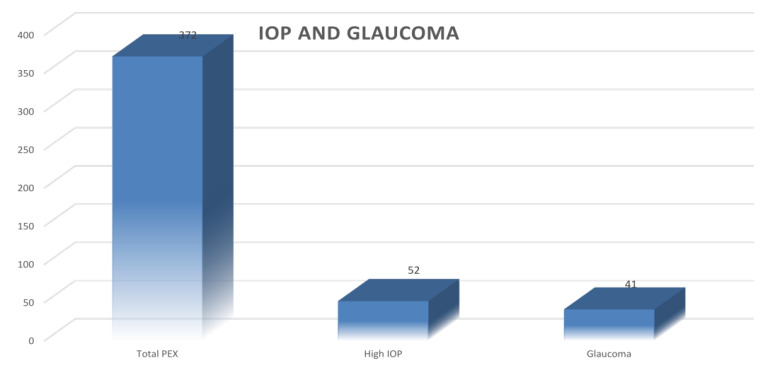
Ocular co morbities among PEX patients

53 cases with pseudoexfoliation (14.2%) had high intraocular pressure and 41 cases (11%) had pseudoexfoliation glaucoma (Figure 8).

## Discussion:

Pseudoexfoliation (PXF) is linked with cataracts and glaucoma, representing the most common form of secondary open-angle glaucoma globally [10]. This study aimed to determine PXF prevalence among patients at a tertiary care teaching institute in Kolar district. The overall prevalence was 10.1% among ophthalmology outpatient attendees from January 2020 to July 2023. The condition predominantly affected one eye and was more prevalent in males. Visual impairment was minimal or absent in most affected eyes, with PXF material frequently found at the pupillary margin. High intraocular pressure was observed in 14.2% of cases, and pseudoexfoliation glaucoma was present in 11% of cases. Previous studies have shown a marked age-related increase in the prevalence of PXF; typically <1% in persons younger than 60 years and increasing to 6.28% among subjects 60 years of age or older. 4 Although the cause of this age-related rise is uncertain, it has been hypothesized that aging-related alterations in gene expression may be responsible. Our study indicates an increasing prevalence of pseudoexfoliation with age, particularly in individuals aged 60 years and older, consistent with findings from the Andhra Pradesh Eye Disease Study and other research by Wariji *et al.* While some studies suggest a higher prevalence among women, it has been suggested that longer life expectancy among women may influence these results. However, other studies have found no significant sex difference. Our study shows a higher prevalence in males, consistent with the Aravind Eye study.

Increased outdoor employment and UV radiation exposure suggest that environmental factors contribute to PXF [4]. It seems to be more common in rural areas, a characteristic that could further enhance the notion of sun radiation[3]. Further, 79.2 % of cases in our study were outdoor workers, a part of the population who are constantly exposed to solar radiation. Data on bilaterality and unilaterality are perplexing with a few studies reiterating that bilateral disease presentations are the most common [5]. Also, 67.7 % of the patients had unilateral affliction in our study. Electron microscopy has revealed deposits of PXF fibrils in the iris muscles, vessels, ciliary body, and trabecular meshwork in cases clinically diagnosed with unilateral disease [3]. Additionally, PXF material has been found in extra-ocular tissues such as the conjunctiva, orbit connective tissue, extra-ocular muscles, vortex veins, and central retinal vessels [4]. This evidence strongly suggests that PXF is primarily a bilateral condition with a likely asymmetric presentation. Therefore, it underscores the importance of thorough bilateral examination, including pupil dilation, in patients initially diagnosed with unilateral disease.

There appears to be a connection between PXF and dementia, Alzheimer's disease, and cognitive impairment [3]. Additional systemic correlations include homocystinuria, aneurysms, ischemic heart disease, hypertension, chronic obstructive pulmonary disease, and cerebrovascular illnesses [9]. Hypertension was documented in 20.9 % of our patients, diabetes mellitus in 16.6 % of patients, asthma in 5.1% of patients, and coronary artery disease in 2.4%. The prevalence in co morbid patients is higher compared with gazelle brue *et al.* study 53 cases (14.2%) with pseudoexfoliation had elevated intraocular pressure (IOP), significantly higher compared to non-PXF cases. Additionally, 41 cases (11%) were diagnosed with pseudoexfoliation glaucoma. Glaucoma prevalence among PXF subjects varies across studies, ranging from 3.0% to 8.3%. It appears more common in older age groups and predominantly affects men consistent with our findings .Our study has several limitations. Firstly, it is retrospective in. The diagnosis of PXF is highly dependent on observer interpretation, which can introduce flaws; cases lacking clinically detectable PXF have been found to exhibit PXF material on conjunctival biopsy [9]. Consequently, this could lead to underestimating the prevalence rates of PXF in the population. As there is no subsequent data available, the number of unilateral cases that eventually progressed to bilateral remains unknown. Because it is a cross-sectional study, the relationship between PXF and glaucoma could not be ascertained, and for this, prospective studies are mandatory. Understanding the prevalence rates of PXF will facilitate the planning of cataract surgery and glaucoma-related eye care services, thereby helping to prevent blindness, especially with the anticipated rise in incidence due to population aging.

## Conclusion:

Data shows that PEX syndrome predominantly affects males in above 60 years old often from lower socio-economic backgrounds and typically manifest unilaterally. Glaucoma was present in 11% of cases, with most eyes experiencing mild or no visual impairment.

## Financial support and sponsorship:

Nil.
